# Neudesin is involved in anxiety behavior: structural and neurochemical correlates

**DOI:** 10.3389/fnbeh.2013.00119

**Published:** 2013-09-09

**Authors:** Ashley Novais, Ana C. Ferreira, Fernanda Marques, José M. Pêgo, João J. Cerqueira, Ana David-Pereira, Filipa L. Campos, Christina Dalla, Nikolaos Kokras, Nuno Sousa, Joana A. Palha, João C. Sousa

**Affiliations:** ^1^Life and Health Sciences Research Institute (ICVS), School of Health Sciences, University of MinhoBraga, Portugal; ^2^ICVS/3B's - PT Government Associate LaboratoryBraga/Guimarães, Portugal; ^3^Department of Experimental Pharmacology, Medical School, University of AthensAthens, Greece

**Keywords:** neudesin, anxiety, dopamine, ventral hippocampus, bed nucleus of the stria terminalis

## Abstract

Neudesin (also known as neuron derived neurotrophic factor, Nenf) is a scarcely studied putative non-canonical neurotrophic factor. In order to understand its function in the brain, we performed an extensive behavioral characterization (motor, emotional, and cognitive dimensions) of neudesin-null mice. The absence of neudesin leads to an anxious-like behavior as assessed in the elevated plus maze (EPM), light/dark box (LDB) and novelty suppressed feeding (NSF) tests, but not in the acoustic startle (AS) test. This anxious phenotype is associated with reduced dopaminergic input and impoverished dendritic arborizations in the dentate gyrus granule neurons of the ventral hippocampus. Interestingly, shorter dendrites are also observed in the bed nucleus of the stria terminalis (BNST) of neudesin-null mice. These findings lead us to suggest that neudesin is a novel relevant player in the maintenance of the anxiety circuitry.

## Introduction

Neudesin (also known as neuron derived neurotrophic factor, Nenf) is a 21 kiloDalton secreted protein with 171 aminoacids (Kimura et al., 2005). Neudesin was classified as a member of the membrane-associated progesterone receptor family since its primary structure contains a cytochrome b5-heme/steroid binding domain (Kimura et al., 2012). Studies in mice showed that neudesin is most abundantly expressed in the brain and spinal cord (Kimura et al., 2005). While in the developing mouse brain neudesin is predominantly expressed in neurons with scattered presence in other cell types, in the adult brain its expression seems to be restricted to neurons (Kimura et al., 2005). Neudesin expression starts at approximately embryonic day 12.5 (E12.5), as evaluated by real time PCR (RT-PCR) in neural precursor cells; its expression increases during the rest of the embryonic period in inverse correlation with the expression of markers of dividing neural precursor cells (nestin) and in direct correlation with that of microtubule-associated protein 2 (MAP-2) (a marker for mature neurons) (Kimura et al., 2005). Of notice, neudesin expression is higher in the cortical preplate, an area that participates in the formation of the cerebral cortex. During embryonic and postnatal development, neurotrophic factors main functions are to provide survival and differentiation of nervous cells through activation of the p75 and tyrosine kinase (Trk) receptors and by downstream pathways such as MAP and PI-3 kinases (Russell and Duman, 2002). In adulthood, these downstream cascades activate different functional responses such as axon growth, dendrite pruning, cell fate decisions (Gray et al., 2013), as well as the modulation of neurotransmitters, thus regulating synaptic plasticity (McAllister, 2001). Importantly, studies in primary cultures of neurons revealed that neudesin display a neurotrophic activity. Specifically, neudesin was shown to induce the proliferation of cortical neural precursor cells early in development, and their subsequent differentiation into neurons (Kimura et al., 2005, 2006). Nevertheless, the identification of a receptor for neudesin is still elusive. Even less information is available on the exact function of neudesin in the adult brain, but *in vitro* experiments indicate that it may promote the maintenance of neurons in an autocrine/paracrine mode (Kimura et al., 2005). This neurotrophic activity has been shown to depend on the attachment of hemin to its cytochrome b5-heme/steroid domain, while a similar relevant action on the binding of steroids failed to be demonstrated (Kimura et al., 2008).

The importance of neurotrophic factors in brain maturation and function was extensively demonstrated (Snider, 1994). Furthermore, it is well-known that neurotrophic factors such as brain derived neurotrophic factor (BDNF) and fibroblast growth factor (FGF) play an important role in the etiology of mood disorders, such as depression, and in modulating emotional responses, including anxiety (Masi and Brovedani, 2011; Turner et al., 2012). In accordance, the limbic brain regions known to be involved in the modulation of emotional responses, that include the ventral hippocampus, amygdala and the bed nucleus of the stria terminalis (BNST), were shown to present an altered neurotrophins levels after an exposure to harmful stimuli, such as chronic stress, thus culminating in neurotransmission imbalance and in synaptic plasticity impairment (Taylor et al., 2011; Jung et al., 2012). Noteworthy, altered monoaminergic neurotransmission was found in BDNF-null mice (Ren-Patterson et al., 2005).

Given the *in vitro* available evidence on the potential neurotrophic properties of neudesin, in this study we addressed the role of Nenf in modulating behavior (emotional and cognitive) brain cytoarchitecture and neurotransmission (namely monoaminergic), for which we used neudesin-null mice.

## Materials and methods

### Animals

A mouse strain with targeted deletion of the Nenf gene, provided by Merck-Serono under a material transfer agreement, was used. The neudesin-null (Nenf^−/−^) mouse strain was generated by using a 129/SvEv genomic library from a BAC clone and the target construct was made by deletion of the entire coding sequence of the neudesin gene (exons 1–4, ~12 KB) and replacing it by a *LacZ-neomicin* cassette. The BAC targeting vector was then inserted into embryonic stem cells (FiH4 ES cells), where the homologous pieces of DNA were recombined. The cells identified as homologous recombinant clones were microinjected into C57BL/6F1 blastocysts to generate chimeric mouse. Initial genotyping was performed using a loss-of-native-allele assay. Animals used in this study were backcrossed in a C57BL/6F background.

Nenf^−/−^ and Nenf^+/+^ were obtained by *crossing heterozygous animals*. Mice genotype was confirmed by PCR using two independent sets of primers: one for the *LacZ* cassette, specific for the genotype: LacZ-foward 5′-GGTAAACTGGCTCGGATTAGGG-3′ and LacZ-reverse 5′-TTGACTGTAGCGGCTGATGTTG-3′; and another for the Nenf gene, specific for Nenf^+/+^ animals: Nenf-intron3 5′- CTTGGAGTTTGGGGCTGATA-3′, Nenf-exon4 5′-TGGCTTTGTACACCTTGCTG-3′. The amplified fragments were of 210 and 176 bp, respectively, and distinguishable by electrophoresis through a 1.5% agarose gel. Confirmation of loss of neudesin synthesis in Nenf^−/−^ was also obtained by performing immunohistochemistry with a neudesin-specific antibody (Sigma, St. Louis, USA) in brain samples of both control and neudesin-null mice; neuronal expression of neudesin was not detected in neudesin-null mice.

Neudesin-null homozygous mice are viable, fertile, grow normally and do not display apparent morphological alterations. Adult animals, at the beginning of behavioral analysis no differences in body weight between littermate controls and neudesin-null mice were found (Nenf^+/+^ = 21.13 ± 1.98 vs. Nenf^−/− = 21.28 ± 2.08 g^).

Animals were maintained under 12 h light/dark cycles at 22 ± 1°C, 55% humidity and fed with regular rodent's chow and tap water *ad libitum*. This study was approved by the Portuguese national authority for animal experimentation, Direcção Geral de Veterinária (permission ID: DGV9457). All experiments were performed in accordance with the guidelines for the care and handling of laboratory animals, as described in the Directive 2010/63/EU of the European Parliament and of the Council.

### Adult behavior

Adult behavior was assessed in 3 months-old male mice, in 3 independent sets of animals, of which one is presented here. Eight neudesin-null and 10 littermates control animals were analyzed in the open field (OF), elevated plus maze (EPM), forced swim test (FST) and Morris water maze (MWM) tests, performed by this sequential order, as an initial behavioral characterization. A 24 h time interval was used between OF, EPM, and FST tests; a 96 h time interval was used between FST and MWM. After the first behavioral characterization, the acoustic startle (AS), light/dark box (LDB) and novelty suppress feeding (NSF) tests were performed, by this sequence, in order to further evaluate the anxious-like behavior (see results section below); for this additional evaluation, another 3 independent sets of animals were analyzed, and one representative set of 10 neudesin-null males and 10 control littermate mice is presented here.

Adult behavior tests were performed in all animals during the light phase of the light/dark cycle at the same period of the day to avoid physiological differences related to the circadian cycle. The tests were performed as described next:

#### Open field

Animals were placed in a room adjacent to the experimental room 1 h before the test. Locomotor activity was assessed in a brightly illuminated square arena with 43.2 × 43.2 cm size surrounded by walls to prevent escape. Animals were placed in the center of the arena and allowed to explore it for 5 min. Data collected through the infrared system (MedAssociates Inc., St Albans, VT) contained total distance travelled, and distance and time spent in the center vs. the periphery of the arena.

#### Forced swim test

Animal learned helplessness behavior was analyzed using the FST in 2 consecutive days. Mice were placed for 5 min in a glass cylinder filled with water (24°C) at a depth of 30 cm. Twenty-four hours later mice repeated the test in the same conditions (Porsolt et al., 1977). Trials were video recorded and manually analyzed using the Etholog V2.2 software (Ottoni, 2000). The 5 min of the second day were analyzed. Data collected consisted in the duration of swimming and of immobility time.

#### Morris water maze

MWM was used to evaluate mice spatial reference memory. In this test, animals were placed in a circular white pool (170 cm in diameter and 50 cm in height) filled with tap water (24 ± 1°C) placed in a poorly lit room. The pool was divided in 4 imaginary quadrants and a transparent plexiglas platform (14 cm in diameter) was hidden 0.5 cm bellow surface in the center of one of the quadrants. For each quadrant, external clues were placed in the walls of the room. The test consisted of 4 trials per day for 4 consecutive days. In each trial, animals were randomly placed in each one of the quadrants and were allowed to swim for 120 s. Mice that failed to reach the platform within this time-period were gently guided to the platform. The distance and time animals took to find the platform were recorded using a video camera connected to a video-tracking system (Videotrack, Viewpoint, Champagne au Mont d'Or, France).

In the fifth day animals performed the probe and reversal tests. The probe test consisted of a unique trial without the platform where the animals were allowed to swim for 120 s. Time and distance swum in each quadrant were collected and analyzed for the first 60 s. To test memory flexibility we performed the reversal test, which consisted in changing the initial position of the platform to the opposite quadrant of the pool. Animals were given 3 trials of 120 s each to learn the new position. The percentage of distance swum in each quadrant is represented.

#### Elevated plus maze

Anxious behavior was assessed using an apparatus composed of two opposite brightly illuminated open arms (51 × 10 cm) and two opposite dark closed arms (51 × 10 × 40 cm) and a central platform, 74 cm above the floor (NIR plus maze, MedAssociates Inc.). Animals were placed in the center of the maze and allowed to explore it for 5 min. Data collected consisted of the number of entries (four paws) in each arm as well as the time spent in each arm (MedPCIV, MedAssociates software).

#### Acoustic startle

Startle reflex was measured in a startle response apparatus (SR-LAB, San Diego Instruments, San Diego, CA, USA), consisting of a non-restrictive plexiglas cylinder (inner diameter 2.8 cm, length 8.9 cm), mounted on a plexiglas platform and placed in a ventilated sound-attenuated chamber. Animals were habituated to the apparatus (5 min) 1 day before actual testing. Cylinder movements were detected and measured by a piezoelectric element mounted under each cylinder. A dynamic calibration system (San Diego Instruments, San Diego, CA, USA) was used to ensure comparable startle magnitudes. Startle stimuli were presented through a high frequency speaker located 33 cm above the startle chamber. Startle magnitudes were sampled every ms over a period of 200 ms, beginning with the onset of the startle stimulus. A startle response is defined as the peak response during 200 ms recording period. A higher startle reflex reflects an increased anxious state of the animal.

#### Light/dark box

For this test the OF arena was divided in half. One part was open and the other consisted of a black plexiglas with an entrance at the center of the arena facing the bright side. Each animal was placed alone at the center of the arena facing the lateral wall and allowed to explore it for 10 min. An infrared system (MedAssociates Inc) registered the time spent in each compartment.

#### Novelty suppressed feeding

Animals were food deprived for 24 h before being placed in the corner of the OF arena (MedAssociates Inc) and left to explore it for 10 min. A single pellet of food was placed in the center of the novel environment and the latency the animal took to leave the corner and feed was recorded. Upon reaching and start eating the pellet the animal was placed back in the home cage, where it was allowed to eat pre-weighted food. Food intake was recorded after 5, 15, and 30 min, as a measure of appetite drive.

### Golgi-cox staining

One week after completion of the behavioral tests mice (5 Nenf^+/+^ and 5 Nenf^−/−^) were transcardialy perfused with 0.9% saline under deep anesthesia and brains were removed for Golgi-Cox staining (Gibbs et al., 1998). Briefly, brains were immersed in Golgi-Cox solution (a 1:1 solution of 5% potassium dichromate (Merck, Darmstadt, Germany) and 5% mercuric chloride (Merck) diluted 4:10 with 5% potassium chromate (Merck) for 14 d; then transferred to 30% sucrose isolution in 0.5% sodium azide where they were kept until processing. Coronal vibrotome 200 μm thick sections were collected in 6% sucrose and blotted dry onto clean, gelatin-coated microscope slides, alkalinized in 18.7% ammonia, developed in Dektol (Kodak, Linda-a-Velha, Portugal), fixed, dehydrated through a graded series of ethanol, cleared in xylene, mounted with entellan and coverslipped.

### Dendritic tree analysis

Basolateral amygdala (BLa) pyramidal neurons, anterior medial bed nucleus of the stria terminalis (amBNST), lateral dorsal bed nucleus of the stria terminalis (ldBNST) bipolar neurons, ventral and dorsal dentate gyrus (DG) granular and ventral CA1 pyramidal-neurons of the hippocampus were chosen randomly, 3 per section; regional boundaries were defined as previously outlined (Dong et al., 2001; Paxinos and Franklin, 2001). The criteria to choose perfect Golgi impregnated neurons were the same as described previously (Uylings et al., 1986): (1) dendritic branches were not incomplete, broken or non-impregnated; (2) dendrites did not show overlap with other branches; (3) neurons were visually well-isolated. Twenty five to 30 neurons per experimental group were studied, i.e., 5–6 neurons per animal were analyzed for each of the 5 animals in the experimental groups. For each selected neuron, all branches of the dendritic tree were reconstructed at 600× magnification using a motorized microscope (BX51, Olympus), with oil objectives attached to a camera (Microbrigthfield Bioscience, Madgedurg, Germany) and using the Neurolucida software (Microbrightfield). Dendritical parameters analyzed were the total dendritic length and 3D version of the Sholl analysis (Sholl, 1956); where, the number of dendritical intersections with concentric spheres positioned at 20 μm intervals from the neurons soma was counted using the NeuroExplorer software (MicroBrightField).

### Neurochemical analysis

Monoamines' levels were measured using high performance liquid chromatography with electrochemical detection (HPLC-ED). Naive 3 months old male mice were killed by decapitation (10 Nenf^+/+^ and 8 Nenf^−/−^). Skulls were snap frozen in liquid nitrogen to avoid degradation during macrodissection. Brains were carefully dissected for ventral hippocampus, amygdala, BNST. Dissection was performed on ice with the help of a stereomicroscope.

Dissected tissues were weighed and then homogenized and deproteinized in 100 μ L of 0.2 N perchloric acid solution (Applichem, Darmstadt, Germany) containing 7.9 mM Na_2_S_2_O_5_ and 1.3 mM Na_2_EDTA (Riedel-de Haën AG, Seelze, Germany), centrifuged at 20000 g for 45 min at 4°C and the supernatant was stored at −80°C until analysis.

The analysis was performed using a GBC LC1150 HPLC pump (GBC Scientific Equipment, Braeside, Victoria, Australia) coupled with a BAS-LC4C (Bioanalytical Systems Inc., USA) electrochemical detector, as previously described (Kokras et al., 2009). The working electrode of the electrochemical detector was set at +800 mV. In all samples reverse phase ion pairing chromatography was used to assay dopamine (DA) and its metabolites 3,4 dihydroxyphenylacetate (DOPAC) and homovanillic acid (HVA), serotonin (5HT) and its metabolite 5-hydroxyindoleatic (5HIAA) and norepinephrine (NE). The mobile phase consisted of a 50 mM phosphate buffer regulated at pH 3.0, containing 5-octylsulfate sodium salt at a concentration of 300 mg/L as the ion pairing reagent and Na_2_EDTA at a concentration of 20 mg/L (Riedel-de Haën AG); acetonitrile (Merck, Darmstadt, Germany) was added at a 9% concentration. The reference standards were prepared in 0.2 N perchloric acid solution containing 7.9 mM Na_2_S_2_O_5_ and 1.3 mM Na_2_EDTA. The column used was an Aquasil C18 HPLC Column, 100 × 1 mm, 5μm Particle Size (Thermo Electron, UK). Samples were quantified by comparison of the area under the curve against known external reference standards using a PC compatible HPLC software package (Chromatography Station for Windows ver.17 Data Apex Ltd). The limit of detection was 1 pg/20 μL (of injection volume). In addition to the assay of 5-HT and 5-HIAA tissue levels, the 5-HT turnover rate was also calculated, separately for each chromatograph, as the ratio of 5-HIAA/5-HT. Similarly, the ratios of DOPAC/DA and HVA/DA were calculated as an index of DA turnover rates. Turnover rates estimate the serotonergic and dopaminergic activities better than individual neurotransmitter and metabolite tissue levels as they reflect 5-HT and DA release and/or metabolic activity as described elsewhere (Dalla et al., 2008; Kokras et al., 2009; Mikail et al., 2012).

### Statistical analysis

All values presented are expressed as the mean ± SEM and significance was verified by using the Mann–Whitney test for independent samples for all behavior, dendritic length, sholl analysis and neurochemical data. Differences were considered significant when *p* < 0.05.

## Results

### Ablation of neudesin induces an anxious-like phenotype in adult mice

Despite the described neuronal expression profile of neudesin in the adult brain, no information is available on the relevance of this protein for the central nervous system (CNS) functioning. To tackle this gap we performed a wide behavioral characterization of neudesin-null mice.

**Figure 1:**
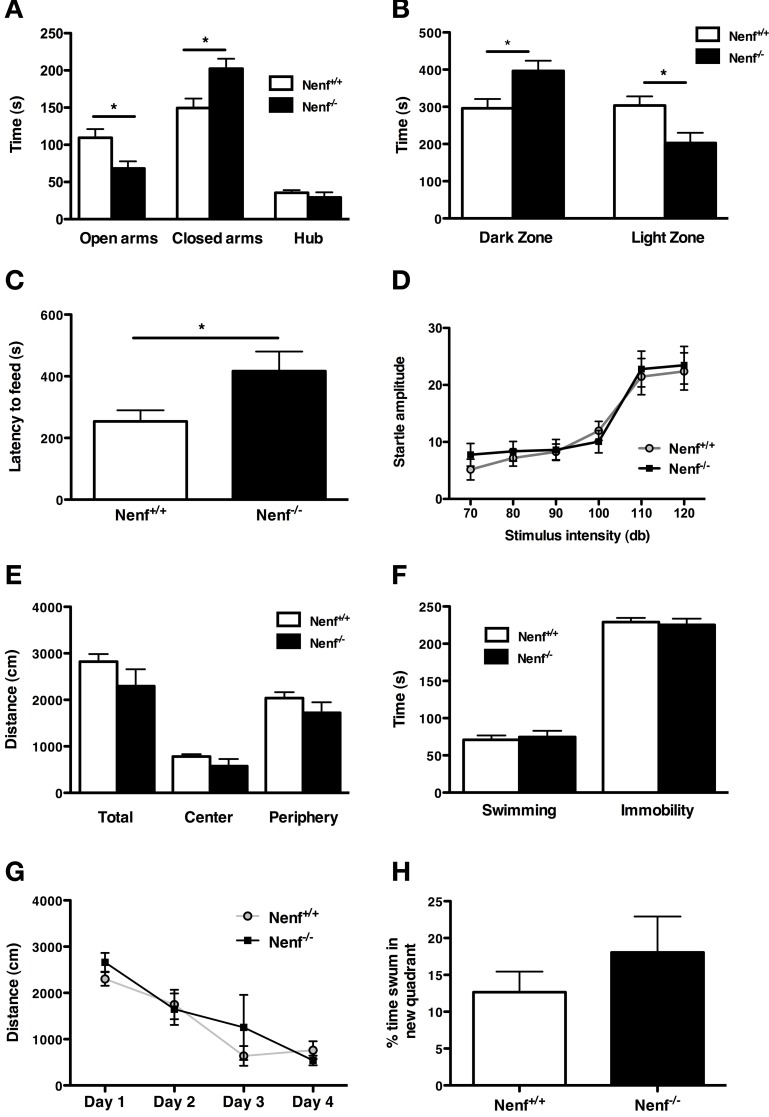
**Neudesin-null mice (Nenf^−/−^) display an anxious-like behavior in contextual paradigms. (A)** Nenf^−/−^ mice spend significantly less time exploring the open arms of the EPM than control mice (Nenf^+/+^). **(B)** The LDB test shows that Nenf^−/−^ mice spend more time in the dark zone than in the light zone. **(C)** Also, in the NSF Nenf^−/−^ mice take longer to feed in the novel environment when compared with Nenf^+/+^. **(D)** The AS test shows that Nenf^−/−^ mice have a similar startle response to higher intensities of sound not reflecting an anxious like behavior in this anxiety dimension. **(E)** In the OF no differences were observed between Nenf^−/−^ and Nenf^+/+^ in the total distance traveled in the arena. **(F)** Performance in the FST, which assessed learned helplessness behavior does not reveal differences between the two groups. **(G)** Cognition, as assessed using the MWM test, is identical between groups, as revealed by the time taken to learn the platform position. **(H)** The % of distance swum in the new quadrant in the reversal trials of the MWM test did not reveal any differences between groups. Data are presented as mean ± SEM. ^*^p < 0.05. OA, open arms; CA, closed arms; H, hub; DZ, dark zone; LZ, light zone?

From the behavioral analyses performed we observed that the ablation of neudesin induces a striking anxious-like phenotype as revealed by a series of tests that specifically assess anxiety-like behavior. We first used the EPM to analyze anxiety-like behavior and found that the percentage of time neudesin-null mice spent in the open arms (22%) was significantly lower than that of controls (37%) (Figure 1A) and, conversely, more time was spent in the closed arms (67%) when compared to controls (50%) (*p* < 0.05). Of relevance, the number of entries in the closed arms (Nenf^+/+^ = 10.8 ± 1.5 and Nenf^−/−^ = 8.5 ± 1.8 s) did not significantly differ between the groups, indicating that the exploratory activity is preserved in neudesin-null mice. This anxious phenotype was further confirmed in other contextual behavioral paradigms, namely the LDB and the NSF. In the LDB test neudesin-null male mice spent more time in the dark zone (396.6 ± 27.5 s) when compared to controls (296.1 ± 25.0 s) (*p* < 0.05) and significantly less time in the light zone (Nenf^+/+^ = 303.4 ± 24.9 vs. Nenf^−/−^ = 202.8 ± 27.5 s) (*p* < 0.05) (Figure 1B); importantly there were no differences regarding the total distance travelled (data not shown). In the NSF test animals have an extra motivational cue—they are fastened for 24 h—so when they are placed in a novel environment, the latency to feed is a measurement of anxiety. In this test we observed that neudesin-null mice displayed increased latency time to eat in the OF arena (416.8 ± 63.6 s) when compared to controls (253.8 ± 35.9 s) (*p* < 0.05) (Figure 1C). The previously described tests are based on conflicts between the animal's innate exploratory/feeding behavior and its aversion for open brightly lit spaces. These are often considered tests for state anxiety and are dependent on cortical processing. On the other hand, the AS test is based on a reflex response to aversive stimuli that tests for a different dimension of anxiety, a more generalized and innate response—trait anxiety. Therefore, a more anxious animal will respond with a bigger startle at higher intensities of sound. Interestingly, the AS test failed to show an anxious like behavior in neudesin-null mice (Figure 1D), even at the higher intensities of sound (120 dB) (Nenf^+/+^ = 22.37 ± 3.26 vs. Nenf^−/−^ = 23.45 ± 3.29 ms) (Figure 1D).

Regarding other behavioral traits, neudesin-null mice did not reveal any phenotype. The OF arena was used to assess locomotor activity. The analysis revealed that neudesin-null mice did not display motor impairments, when compared to control animals (Figure 1E). We used the FST to measure helplessness behavior, a behavioral dimension relevant for depression. In the FST (Figure 1F) both controls and neudesin-null animals spent a similar amount of time immobile (Nenf^+/+^ = 229 ± 5 vs. Nenf^−/−^ = 225 ± 8 s), thus indicating that neudesin-null mice do not display a depressive-like behavioral phenotype. Cognition, specifically spatial reference memory, was analyzed in the MWM. Both neudesin-null and control mice learned the position of the hidden platform as they similarly decreased the latency of time required to perform the task, indicating an absence of cognitive impairment (Figure 1G); this was further confirmed in the probe test (data not shown). Regarding performance in the reverse learning task of the MWM, the percentage of distance swum in the new quadrant by neudesin-null mice was similar to that of control mice (Figure 1H).

### Neudesin-null adult male mice have altered neuronal morphology

**Figure 2:**
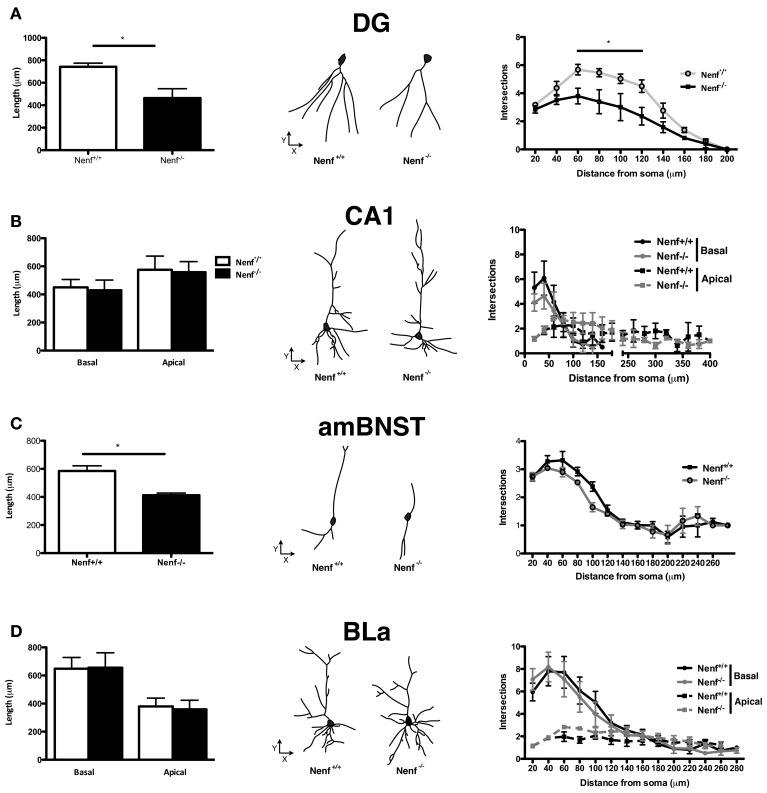
**Three-dimensional morphometric analysis of Golgi-Cox impregnated neurons of the ventral hippocampus, BNST and amygdala.** Nenf^−/−^ mice have shorter dendrites than controls in the dentate gyrus (DG) of the ventral hippocampus (**A**, left panel) as well as less intersections between dendrites (**A**, right panel). These differences in dendrite morphology and neuron arborization are not observed for the hippocampal CA1 region (**B**, left and right panel, respectively). Neudesin-null mice have shorter dendritic length in the anterior medial division of the BNST (amBNST) and no differences in the number of intersections in the sholl analysis (**C**, left and right panel, respectively). Analysis of the basolateral nucleus of the amygdala (BLa) revealed no differences concerning dendritic length or arborization (**D**, left and right panels, respectively). The middle panels correspond to a representative 3D reconstructed neuron from each region analyzed from both Nenf^+/+^ (left) and Nenf^−/−^ (right). Data are presented as mean ± SEM. ^*^p < 0.05.

Neuronal morphology, dendrite formation and the establishment of synaptic contacts are dependent of trophic support. Due to the potential role of neudesin as a neurotrophic factor we studied the 3D-morphology of neurons in neudesin-null mice. In light of the anxiety-like phenotype described before, we focused the analysis in brain regions implicated in the modulation of this behavioral trait, namely the ventral hippocampus, the amygdala (BLa nuclei) and the BNST. When compared to control mice, neudesin-null mice presented shorter dendritic length of ventral hippocampal DG granular neurons (Nenf^+/+^ = 741.8 ± 32.8 vs. Nenf^−/−^ = 463.9 ± 83.3 μm) (*p* < 0.05) (Figure 2A left panel). Sholl analysis also revealed fewer intersections of the dendritic tree (with the imaginary spheres) at distances between 60 and 120 μm from the soma in neudesin-null mice (*p* < 0.001) (Figure 2A right panel). Nevertheless, in ventral hippocampal CA1 pyramidal-like neurons no difference was found between control and neudesin-null mice in dendritic length or dendritic arborization, both for the basal and for the apical dendrites (Figure 2B). Noteworthy the differences found for ventral hippocampal DG neurons were not observed in the dorsal DG granular neurons (Nenf^+/+^ = 644.7 ± 29.3 vs. Nenf^−/−^ = 606.3 ± 39.9 μm). Regarding the BNST, two different divisions were analyzed, the anterior medial BNST (amBNST) and the lateral dorsal BNST (ldBNST); neudesin-null mice had a statistically significant reduction in dendritic length in the anterior medial (Nenf^+/+^ = 584.8 ± 36.7 vs. Nenf^−/−^ = 412.6 ± 14.7 μm) (Figure 2C left panel), but no differences in the lateral dorsal division (Nenf^+/+^ = 628.7 ± 64.5 vs. Nenf^−/−^ = 485.2 ± 34.1 μm), as well as no differences regarding dendritic arborization of neurons in both BNST divisions. Finally, the morphology of pyramidal-like neurons of the BLa was analyzed and no differences were observed between control and neudesin-null mice (Figure 2D).

### Neurotransmitter activity profile in anxiety related regions

Given the behavioral and structural alterations found in neudesin-null mice we next characterized the monoaminergic profile (major findings presented Table 1 for complete data) of several anxiety-related brain regions (amygdala, BNST, ventral hippocampus). We observed a reduction of DA levels in the ventral hippocampus of neudesin-null animals (*p* < 0.05) (Table 1), which was accompanied by an elevated dopaminergic turnover of HVA/DA (*p* < 0.05) (Table 1). Regarding the amygdala, there was a trend for a reduction in DA, DOPAC, and NE levels (Table 1); in the BNST no alterations were observed in monoamines levels or turnover (Table 1).

**Table 1:** **Levels of monoamines in brain areas of littermate control (Nenf^+/+^) and neudesin-null (Nenf^−/−^) mice**.

	**Nenf^+/+^**	**Nenf^−/−^**
**VENTRAL HIPPOCAMPUS**		
NE	1.49 ± 0.22	1.06 ± 0.31
DA	0.49 ± 0.10	0.27 ± 0.09^*^
DOPAC	0.43 ± 0.15	0.35 ± 0.15
HVA	0.32 ± 0.06	0.27 ± 0.10
DOPAC/DA	0.78 ± 0.13	1.17 ± 0.22
HVA/DA	0.61 ± 0.07	1.02 ± 0.08^**^
5HT	8.69 ± 1.51	5.21 ± 1.31
5HIAA	10.59 ± 2.40	6.28 ± 1.92^*^
5HIAA/5HT	0.94 ± 0.09	1.08 ± 0.09
**BED NUCLEUS OF THE STRIA TERMINALIS**		
NE	9.44 ± 1.49	13.58 ± 4.33
DA	6.77 ± 1.51	9.54 ± 3.85
DOPAC	8.32 ± 2.57	9.89 ± 3.95
HVA	5.38 ± 1.15	6.52 ± 1.85
DOPAC/DA	0.96 ± 0.10	1.33 ± 0.20
HVA/DA	0.72 ± 0.10	1.16 ± 0.21
5HT	13.12 ± 3.69	16.12 ± 4.88
5HIAA	6.72 ± 1.41	9.80 ± 2.35
5HIAA/5HT	0.60 ± 0.06	0.72 ± 0.07
**AMYGDALA**		
NE	2.50 ± 0.69	1.57 ± 0.41
DA	6.76 ± 1.56	3.00 ± 1.09
DOPAC	7.54 ± 2.40	3.16 ± 0.86
HVA	3.33 ± 1.04	1.38 ± 0.35
DOPAC/DA	0.99 ± 0.14	1.38 ± 0.45
HVA/DA	0.50 ± 0.07	0.56 ± 0.12
5HT	15.08 ± 3.31	6.50 ± 0.98
5HIAA	10.15 ± 2.81	5.97 ± 1.58
5HIAA/5HT	0.64 ± 0.07	0.91 ± 0.21

Levels of catecholamines and monoamines are in μg/g tissue and expressed as means ± S.E.M.

* p < 0.05 and

** p < 0.01.

Regarding serotonin, a significant reduction of the serotonin metabolite 5HIAA (*p* < 0.05) (Table 1) was seen in the ventral hippocampus, but this difference did not translate into any alterations regarding serotonin turnover. An ~40–50% reduction in the levels of both 5HT and 5HIAA in neudesin-null mice was also observed in the amygdala but these differences did not reach statistical significance for both metabolites. In the BNST no differences were observed for 5HT metabolites and respective turnover rates.

## Discussion

This work provides the first *in vivo* demonstration of the relevance of the neurotrophic factor neudesin for CNS normal function. In the absence of neudesin, mice display a contextual anxious like phenotype that is accompanied by impairment in the dopaminergic activity of the ventral hippocampus, where the dendritic arborization in granular neurons branching is impoverished; in addition, they also present a dendritic atrophy in the amBNST nucleus.

We first addressed the role of neudesin by exploring the behavioral consequences of its ablation. Of the several behavioral dimensions assessed, only anxiety-related phenotypes were clearly affected by the absence of neudesin, as demonstrated by the shorter time spent in the open arms of the EPM and in the light zone of the LDB and the higher latency to eat in the NSF test. Interestingly, no deficits were found in the AS, which reveals that the increased anxiety occurs specifically in contextual conflict paradigms, described to involve modulation by different neuronal circuits (Koch and Schnitzler, 1997). Anxiety in the presence of contextual anxiogenic environments, such as those observed here, is shown to be mediated by the BNST (Ventura-Silva et al., 2012) under the modulation of different cortical regions namely the hippocampus and the prefrontal cortex (Ventura-Silva et al., 2013). The BNST is closely involved in stress and in the HPA axis-dependent modulation of emotional behaviors (Herman and Cullinan, 1997). It has been reported that anxiety triggered by different stress-inducing paradigms is related to hypertrophy of the amBNST neurons (Pego et al., 2008; Oliveira et al., 2012). Surprisingly, however, neudesin-null mice display shorter dendrites in the amBNST, which suggests this might be a stress-specific effect and that other factors beyond the pure structure of these neurons might underlie the behavioral changes displayed by neudesin-null mice.

Interestingly, the same holds true for the ventral hippocampus, where we also found dendritic atrophy, specifically in granule cells. The role of the ventral hippocampus in anxiety has been a matter of debate, with some studies showing that an increased activity in this brain region is related with increased emotionality (McHugh et al., 2004, 2011), while others studies do not confirm this association (Marrocco et al., 2012). Recently, however, the activity of ventral granule cells has been specifically implicated in anxiety behavior. Using optogenetics tools to activate and to inhibit DG granular cells in the ventral hippocampus, Fournier et al saw that elevating activity in this area suppresses anxiety in the EPM (Fournier and Duman, 2013). The present results seem to support the later study, in as much as we show that neudesin-null mice present an impoverished arborization in granule cells of the ventral hippocampus. Noteworthy, this later effect is specific to the ventral hippocampus since we found no differences between control and neudesin-null mice in the arborization of dorsal DG granular cells. Given the recently shown role of dorsal DG cells in the modulation of learning (Kheirbek et al., 2013), this result is in accordance with the absence of a cognitive phenotype in neudesin-null mice. This specific alteration in the atrophy of DG granule cells along the dorsal-ventral axis in the absence of neudesin is of relevance and deserves further investigation.

Going further into the molecular levels, we next studied the monoaminergic profile of anxiety-related brain regions. Interestingly, the absence of neudesin is associated with low levels of DA and increased HVA/DA turnover ratio in the ventral hippocampus. Importantly, DA levels are described to be higher in the ventral than in the dorsal hippocampus (Eisenhofer et al., 2004) and DA is known to play a crucial role in modulating plasticity in the ventral portion of the hippocampus (Belujon and Grace, 2011). In addition, dopamine 1 receptor (D1) is highly expressed in the dendrites of the granular cells of the ventral DG (Mansour et al., 1992), and dopamine release from projecting ventral tegmental area neurons impacts on the modulation of synaptic plasticity and synapse strenght in this brain region (Hamilton et al., 2010). Whether decreased DA release in the ventral hippocampus, as observed in neudesin-null mice, contributes to the dendritic atrophy in ventral DG granular neurons is unknown. Nevertheless, the role of dopamine in anxiety is also endowed in controversy: while pioneer studies with systemic apomorphine (a D1/D2 agonist) administration were shown to decrease anxiety (Hjorth et al., 1986), more recent studies using specific administration of this drug in the ventral hippocampus increased anxiety in the EPM (Zarrindast et al., 2010). Regarding the levels of 5HT, it is of notice that the ventral hippocampus of neudesin-null mice displays a decrease (although not significant) of 40% in the levels of 5HT and a sharp reduction in the levels of its derived metabolite 5HIAA. This is of relevance since selective knock-down of auto 5HT-1A receptors in the raphe nuclei of mice results in a direct increase of anxiety levels in contextual paradigms, and in a concomitant decrease in the extracellular levels of 5HT in the ventral hippocampus (Richardson-Jones et al., 2011). Thus, the alteration in serotonergic metabolites observed in the ventral hippocampus may contribute to the anxiety state observed in neudesin-null mice, in accordance to previous studies in stressed animals (Dalla et al., 2008). Also interesting, although not significant, is the reduction in the 5HT levels in the amygdala of neudesin-null mice. This may be of relevance when considering the fundamental role of 5HT as a player in the crosstalk between amygdala and the ventral hippocampus in the modulation of anxiety behavior (Asan et al., 2013). Overall, these results further highlight a possible role for neudesin in the establishment and/or maintenance of hippocampal circuitry and in the modulation of contextual anxiety-behavior.

Both during embryonic and postnatal brain development several external signals including neurotrophic factors, neurotransmitters and hormones are involved in the genesis and maturation of new neurons and in their integration into functional circuitries (Abrous et al., 2005). One of the unique roles previously ascribed to neudesin, as shown *in vitro*, is its neurotrophic activity (Kimura et al., 2005, 2006). Thus, it is plausible that the herein described functional alterations in neuronal arborization result from the absence of neurotrophic support conveyed by neudesin. On the other hand, neudesin structure displays a heme and/or steroid-binding site (Kimura et al., 2005), which seems necessary for its function (Kimura et al., 2008). Free heme is a powerful oxidative stressor (Jeney et al., 2002; Craven et al., 2007; Abraham and Kappas, 2008) and under physiological conditions it is bound to extracellular heme binding proteins such as Nenf. Thus, we cannot discard the possibility that the deleterious effects of free heme due to the absence of neudesin might have implications in the maintenance of neurons as previously suggested (Burmester and Hankeln, 2004).

The data presented in this study suggest that neudesin modulates anxiety behavior mainly through the DG ventral hippocampus and altered dopaminergic activity. Thus, the neurotrophic action described previously for neudesin per ser, or in association with its ligands, might be of therapeutical and/or pharmacological potential in anxiety-related disorders. The modulatory role of neudesin in anxiety might be associated with its putative neurotrophic role but why this effect is specific for the anxiety circuits is still to be determined. Nevertheless, since the mouse model used in this study is a constitutive knock-out of the Nenf gene, we cannot exclude potential developmental determinants (Stevens et al., 2010) resulting from the absence of neudesin in adult anxiety circuits, which should next be investigated.

### Conflict of interest statement

The authors declare that the research was conducted in the absence of any commercial or financial relationships that could be construed as a potential conflict of interest.
